# Enhanced Neuroplasticity by the Metabolic Enhancer Piracetam Associated with Improved Mitochondrial Dynamics and Altered Permeability Transition Pore Function

**DOI:** 10.1155/2016/8075903

**Published:** 2016-09-26

**Authors:** Carola Stockburger, Davide Miano, Thea Pallas, Kristina Friedland, Walter E. Müller

**Affiliations:** ^1^Department of Pharmacology, University of Frankfurt and Biocenter, Goethe University, 60438 Frankfurt, Germany; ^2^Molecular and Clinical Pharmacy, Department of Chemistry and Pharmacy, Friedrich-Alexander-Universität Erlangen-Nuremberg, 91058 Erlangen, Germany

## Abstract

The mitochondrial cascade hypothesis of dementia assumes mitochondrial dysfunction leading to reduced energy supply, impaired neuroplasticity, and finally cell death as one major pathomechanism underlying the continuum from brain aging over mild cognitive impairment to initial and advanced late onset Alzheimer's disease. Accordingly, improving mitochondrial function has become an important strategy to treat the early stages of this continuum. The metabolic enhancer piracetam has been proposed as possible prototype for those compounds by increasing impaired mitochondrial function and related aspects like mechanisms of neuroplasticity. We here report that piracetam at therapeutically relevant concentrations improves neuritogenesis in the human cell line SH-SY5Y over conditions mirroring the whole spectrum of age-associated cognitive decline. These effects go parallel with improvement of impaired mitochondrial dynamics shifting back fission and fusion balance to the energetically more favorable fusion site. Impaired fission and fusion balance can also be induced by a reduction of the mitochondrial permeability transition pore (mPTP) function as atractyloside which indicates the mPTP has similar effects on mitochondrial dynamics. These changes are also reduced by piracetam. These findings suggest the mPTP as an important target for the beneficial effects of piracetam on mitochondrial function.

## 1. Introduction

Mitochondrial dysfunction is one of the major mechanisms underlying brain aging, mild cognitive impairment (MCI), and late onset Alzheimer's disease (LOAD) [1–6]. The velocity of the decline of mitochondrial function depends on individual genetic predisposition like APOE4 and environmental factors [4, 7] until mitochondrial energy production falls below a critical threshold [1, 2]. Exceeding this threshold may lead to conditions where mitochondrial dysfunction gets further exaggerated by the combined effects of aging, mildly elevated amyloid-*β* (A*β*) levels formation due to the stimulation of gamma-secretase activity [8, 9], and increased free radical (ROS) formation. Thus, mitochondrial dysfunction represents a major player within the interface between aging and AD [8–12]. As a result, mitochondrial dysfunction associated with reduced energy supply and enhanced free radical (ROS) formation finally leads to impaired neuroplasticity including reduced neuritogenesis and neuronal cell loss [11–13]. Thus, improving mitochondrial dysfunction has become an important strategy for the development of drugs to treat the earlier stages of cognitive decline [5, 11, 12, 14–17]. One example is the metabolic enhancer piracetam, the prototype of the so-called “nootropic” drugs [18]. Piracetam has been shown to improve impaired cognitive functions in various conditions in man and in animal models using different conditions of impaired brain function [18–23]. Even if its clinical efficacy is seen controversially, piracetam is still used in many countries to treat cognitive impairment in aging and dementia, following brain injuries and stroke, as well as in mild to moderate dementia [19–23].

Our and others' previous studies indicated that piracetam shows neurotrophic and neuroprotective effects by ameliorating impaired mitochondrial function (enhanced mitochondrial membrane potential and elevated ATP production) [24–27]. While some initial findings suggested that these effects might also be associated with effects on mitochondrial dynamics, the exact relationship between both parameters remained unclear [28]. Accordingly, we investigated in the experiments outlined in the present communication effects of piracetam on neuritogenesis in the human neuronal cell line SH-SY5Y [29, 30] showing a possible relationship to mitochondrial fission and fusion balance (dynamics) and to the function of the mitochondrial permeability transition pore (mPTP).

## 2. Materials and Methods

### 2.1. Materials

Dulbecco's Modified Eagle Medium, OptiMEM® Reduced Serum Medium, hygromycin, penicillin, streptomycin, MEM Vitamin solution, MEM Nonessential Amino Acids, sodium pyruvate, and Mito Tracker CMXRos were purchased from Invitrogen, Karlsruhe, Germany. All chemicals were obtained from Sigma Aldrich, Hamburg, Germany, unless otherwise stated. For ATP determination, the ViaLight*™* Plus Cell Proliferation and Cytotoxicity BioAssay Kit from Lonza, Basel, Switzerland, was used. Antibodies were purchased from Millipore, Billerica, USA [anti-GAP43 (MAP347), anti-Glyceraldehyde-3-Phosphate Dehydrogenase (MAB374), and all secondary antibodies].

### 2.2. Cell Culture

PC12 cells were cultured in DMEM supplemented with 10% heat-inactivated fetal calf serum and 5% heat-inactivated horse serum, 60 units/mL penicillin, and 60 *µ*g/mL streptomycin at 37°C in a humidified incubator containing 5% CO_2_.

SH-SY5Y cells and SH-SY5Y APPwt cells stably transfected with DNA constructs containing the entire coding region of human A*β*PP (A*β*PP695) or the corresponding vector alone (pCEP4 vector) were kindly donated by A. Eckert (Basel, Switzerland) [31]. Cells were cultured in Dulbecco's Modified Eagle Medium supplemented with 10% heat-inactivated fetal calf serum, 0.3 mg/mL hygromycin, 60 units/mL penicillin, 60 *µ*g/mL streptomycin, MEM Vitamin solution, MEM Nonessential Amino Acids, and 1 mM sodium pyruvate at 37°C in a humidified incubator containing 5% CO_2_.

### 2.3. Animals

12-week-old and 18-month-old female Naval Medical Research Institute (NMRI) mice used in this study were purchased from Charles River (Borchen, Germany). The latter were obtained at an age of 12 months and maintained at the Biocenter's animal care facility until use. For western blot analysis, isolated hippocampus and frontal cortex were used. To obtain isolated mitochondria for mitochondrial swelling experiments, whole brain homogenates were used. All animals were housed in plastic cages with water and food* ad libitum* and were maintained on a 12 h light/dark cycle. Animals were handled and killed for “*ex vivo*” experiments according to the German and University Frankfurt guidelines for animal care.

### 2.4. Neurite Length

For determining neurite length, all cells were seeded on polylysine coated glass cover slips and treatment was started one day after seeding. PC12 cells were grown in 15% serum containing medium overnight. The next day, medium was changed to a medium containing 2% serum and nerve growth factor (NGF, 50 ng/mL) to induce differentiation. SH-SY5Y cells were seeded and incubated with normal 10% serum containing cell culture medium for 24 hours. The next day, media were changed to reduced cell culture medium containing 2% serum. All cells were fixed with phosphate buffered formalin solution (4%) for 20 minutes and stained with hematoxylin and eosin solutions. 30 cells from each stain were arbitrarily investigated and neurite length was detected by using Nikon NIS Elements AR 2.1 software.

### 2.5. Western Blot

Cells were washed with phosphate buffered saline (PBS) and lysed in lysis buffer containing 1 mM ethylenediaminetetraacetic acid, 5 mM sodium fluoride, 0.5% Triton*™* X-100, 6 M urea, 0.5% sodium deoxycholate, 0.5% sodium dodecyl sulfate, 2.5 mM sodium pyrophosphate, 1 mM sodium orthovanadate, 3 *µ*g/mL aprotinin, 0.01 mg/mL leupeptin, 0.01 mg/mL pepstatin, and 0.1 mM phenylmethanesulfonyl fluoride (PMSF) in PBS. After protein determination (bicinchoninic acid method), 10 *μ*g of protein per lane was loaded on a 4–12% Bis-Tris gel and separated by electrophoresis. Samples were transferred onto a polyvinylidene fluoride membrane and incubated for 1 hour with blocking solution, washed three times with Tris buffered saline with Tween® solution (TBST), and incubated with primary antibodies overnight at 4°C. After washing five times with TBST, membranes were treated with horseradish peroxidase conjugated secondary antibodies, washed five times with TBST, and analyzed using a ChemiDocXRS system (Bio Rad, Munich, Germany).

### 2.6. ATP Levels

ATP levels were determined using a bioluminescence assay based on a luciferase reaction which catalyses the formation of light from ATP and luciferin. 2 × 10^4^ cells were seeded in a white walled 96-well plate and treated according to the manufacturer's instructions. The emitted light is linear to the ATP concentration and was measured with a VICTOR*™* X2 Multilabel Plate Reader (Perkin Elmer).

### 2.7. Mitochondrial Membrane Potential

SH-SY5Y cells were plated two days before measurement at a density of 1 × 10^5^ cells per well in a 24-well plate. Cell media were incubated with 0.4 *µ*M Rhodamine 123 (R123) for 15 minutes and afterwards washed twice with 500 *µ*L Hank's Balanced Salt Solution (pH 7.4, 37°C). The fluorescence was measured using a VICTOR X2 Multilabel Plate Reader (Perkin Elmer) at 490/535 nm. The transmembrane distribution of the fluorescence dye R123 depends on the MMP and therefore is proportional to its strength.

### 2.8. Mitochondrial Shape Quantification Using Confocal Laserscan Microscopy

SH-SY5Y cells were seeded on polylysine coated glass cover slips for 4 days. Mitochondria were visualized by labelling with MitoTracker® Deep Red FM (100 nM) for 2 hours. Cells were fixed with phosphate buffered formalin solution (4%, 37°C, pH 7.4) for 20 minutes and washed three times with PBS. The samples were analyzed using a Leica TCS SP5 confocal laserscan microscope with a 63x oil immersion objective and Image J 1.47t (National Institute of Health, USA). Mitochondrial shape was quantified by separating four groups: punctuated (0–2 *µ*m), truncated (2–4 *µ*m), tubular (4–10 *µ*m), and elongated (>10 *µ*m length); *n* = 100 mitochondria.

### 2.9. Mitochondrial Swelling

The possible effects of piracetam on mitochondrial swelling in brain mitochondria were evaluated in “*ex vivo*” experiments by measuring spectrophotometric alterations in light scattering according to Hansson et al. [32] with slight modifications. Isolation of mitochondria was achieved using a Percoll gradient according to Sims and Anderson [33] and Hansson et al. [32] with slight modifications. Preparation was carried out in ice cold solutions. In brief, NMRI mice were decapitated, brains were quickly dissected on ice, and, after removing the cerebellum, brains were washed with isolation buffer (320 mM sucrose, 2 mM EGTA, 10 mM Trizma base, and pH 7.4). Brains were homogenized in isolation buffer containing 12% Percoll using a Tissue Grinder Dounce (Wheaton, Millville, USA) by ten loose and ten tight strokes. Afterwards, the homogenates were slowly layered directly onto previously prepared discontinuous Percoll gradients (26% Percoll layered above 40% Percoll) and centrifuged in a Beckmann J2-HS and rotor J20.1 (30,700 ×g, 7 min, 4°C). The mitochondrial fraction was removed (band 3; for details, see [34]), diluted with isolation buffer, and centrifuged (16,700 ×g, 12 min, 4°C). The resulting pellet was washed twice with isolation buffer (7,300 ×g, 6 min, 4°C) and total protein content was estimated (Bradford method). Mitochondria were diluted with isolation buffer to a total protein content of 2.75 mg/mL. Mitochondrial permeability transition was monitored by measuring the decrease in 90° light scattering at 520 nm (emission and excitation) using a Aminco Bowman Series 2 Spectrometer (SLM-Aminco, Rochester, USA) over 750 seconds as described by Stockburger et al. [30]. Mitochondria (27.5 *µ*g) were incubated in a stirred glass cuvette containing 1.1 mL measuring buffer (250 mM sucrose, 10 mM Trizma base, 20 mM MOPS, 2 mM KH_2_PO_4_, 1 mM MgCl_2_, 1 *µ*M EGTA, and pH 7.2) with glutamate (5 mM) and malate (5 mM) for three minutes. Afterwards, oligomycin (1 *μ*g/mL) was added and the measurement was started. After 60 seconds, either the inhibitor of mPTP formation cyclosporine A (1 *μ*M) or ethanol (absolute) as solvent control or piracetam was added. After further 60 seconds, ADP (20 *μ*M) was added. Swelling was induced by the addition of calcium (1 *μ*mol/mg protein) or atractyloside (400 *µ*M) after 300 seconds. Alamethicin (16 *μ*g/mL) was added after 500 seconds to induce maximal mitochondrial swelling. The absorbance before calcium injection was set as 0% and the absorbance after alamethicin injection as 100%. The quality of mitochondrial purification was verified by measuring the increase in mitochondrial respiration after adding cytochrome c (10 *µ*M) and by determining the respiratory control ratio (all mitochondrial preparations with an RCR less than 4 were discarded).

### 2.10. Statistical Analysis

Data are given as mean ± SEM. For statistical comparison, Student's *t*-test and Two-Way ANOVA followed by Bonferroni posttest for multiple comparisons were used.* p* values < 0.05 were considered statistically significant. “*n*” stands for the number of independent experiments carried out.

## 3. Results

### 3.1. Piracetam Improves Synaptic Plasticity after Mitochondrial Impairment

**Figure 1:**
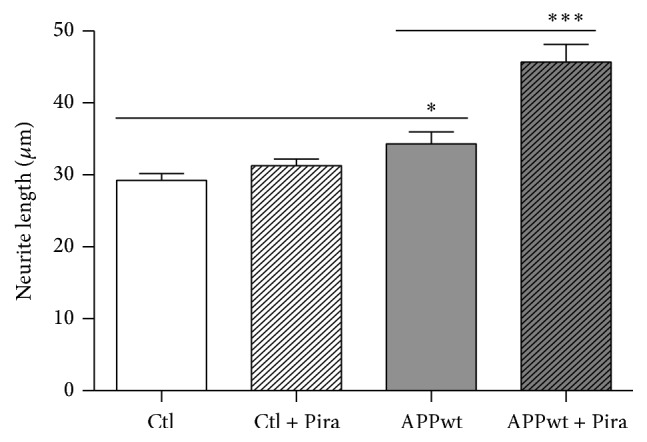
Piracetam improves neurite outgrowth under normal conditions in SH-SY5Y APPwt cells only. SH-SY5Y Ctl cells were seeded and incubated for one day. Afterwards, incubation was started with 1 mM piracetam (Pira). After 72 hours, cells were fixed and stained for analysis of neurite outgrowth. In SH-SY5Y Ctl cells, no statically significant effect was observed. In contrast, SH-SY5Y APPwt cells benefit from the incubation with piracetam and possess longer neurites. Data are represented as mean ± SEM; Student's unpaired *t*-test (^*∗*^ *p* < 0.05, ^*∗∗∗*^ *p* < 0.001 compared to untreated control cells); *n* = 8–14.

**Figure 2:**
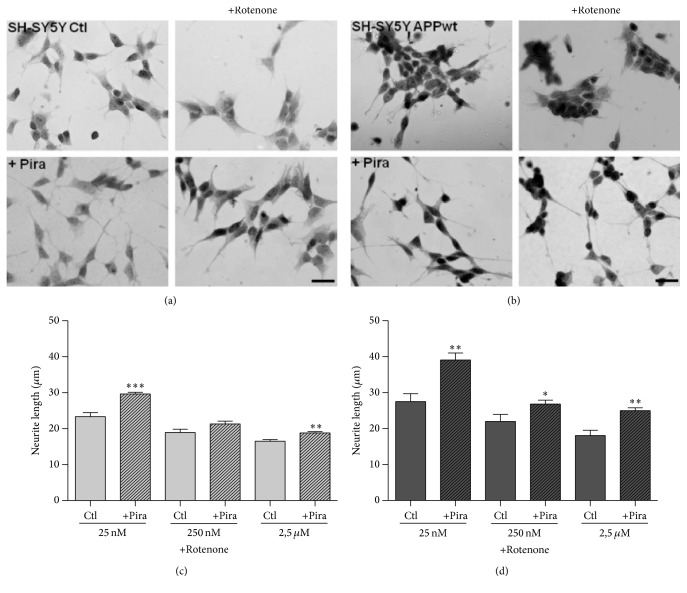
Piracetam diminishes changes in neurite outgrowth after complex I impairment. (a) Representative pictures of SH-SY5Y Ctl and (b) SH-SY5Y APPwt cells treated with 250 nM rotenone and piracetam (Pira); scale bar represents 25 *µ*m. (c) SH-SY5Y Ctl and (d) SH-SY5Y APPwt cells were seeded and incubated for one day. Afterwards, incubation was started with 1 mM piracetam (Pira) for 72 hours. During the last 24 hours, cells were additionally treated with different concentrations of rotenone (25 nM, 250 nM, or 2.5 *µ*M). Afterwards, cells were fixed and stained for analysis of neurite outgrowth. Data are represented as mean ± SEM; One-Way ANOVA with Tukey's Multiple Comparison test (^*∗*^ *p* < 0.05, ^*∗∗*^ *p* < 0.01, and ^*∗∗∗*^ *p* < 0.001 compared to rotenone treated cells); *n* = 6–14.

**Figure 3:**
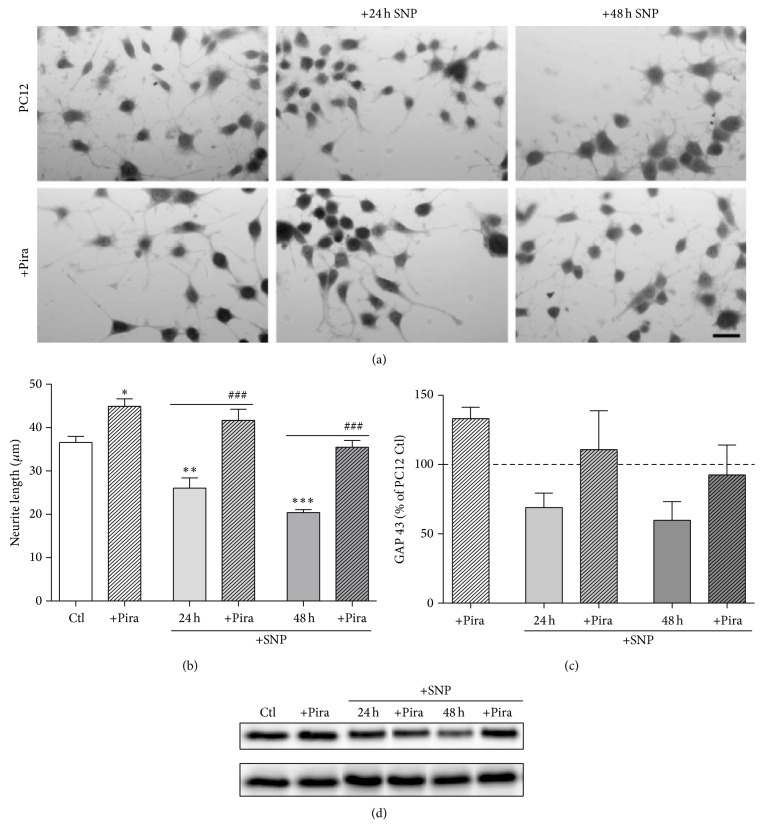
Piracetam improves neurite outgrowth under normal conditions and after oxidative stress in PC12 cells. (a) Representative pictures of PC12 cells treated with 50 *µ*M sodium nitroprusside (SNP) for 24 or 48 hours and 1 mM piracetam (Pira). (b) PC12 cells treated with piracetam (Pira) exhibit significantly longer neurites. When cells were additionally treated with SNP, piracetam (Pira) is able to diminish neurite shortening. (c) In parallel, PC12 cells treated with piracetam possess slightly elevated GAP43 levels. PC12 cells were differentiated with nerve growth factor (50 ng/mL) for 72 hours and were incubated with piracetam (1 mM) in parallel. SNP (50 *µ*M) was added for the last 24 or 48 hours, respectively. Cells were fixed for analysis of neurite outgrowth or harvested for western blot analysis. (d) Representative western blot analysis of GAP43 in PC12 cells treated with different concentrations of Pira and/or SNP. GAPDH was used as loading control. Data are represented as mean ± SEM; One-Way ANOVA with Tukey's Multiple Comparison test (^*∗*^ *p* < 0.05, ^*∗∗*^ *p* < 0.01, and ^*∗∗∗*^ *p* < 0.001 compared to untreated control cells; ^###^ *p* < 0.001 compared to SNP treated cells); *n* = 6–10.

For assessing possible effects of piracetam on mechanisms of synaptic plasticity, we used SH-SY5Y cells, a human neuronal cell line as an established cell model to track changes in synaptic plasticity [29, 30, 35–37]. Under normal conditions of tissue culture without additional growth factors, SH-SY5Y cells already show substantial neuritogenesis [29]. Interestingly, under these conditions, piracetam had no effect on neurite outgrowth (Figure 1), which parallels many other observations with piracetam that beneficial effects are mainly present for conditions of impaired neuronal function [18]. We have previously shown that the SH-SY5Y cells can also be used to mirror the continuum of mitochondrial impairment from aging to the initial and the later stages of late onset Alzheimer's disease (LOAD) [1, 2] by the addition of the complex I inhibitor rotenone as a model for the brain aging process, by slightly elevated *β*-amyloid (A*β*) levels due to the transfection with an additional copy of the human APP gene (SH-SY5Y APPwt) for the initial phase and by the combination of both interventions for the advanced phase of LOAD [29]. For all three conditions, piracetam showed a substantial improvement of neuritogenesis (Figures 1 and 2). To confirm the association of increased neurite length following piracetam treatment with elevated levels of the presynaptic protein GAP43, we had to switch again to PC12 cells [25] since GAP43 was already present in SY5Y cell bodies at very high levels. In line with our preliminary findings, treating differentiating PC12 cells with piracetam elevates significantly neurite outgrowth and increases the expression of the synaptic marker GAP43 by trend (Figure 3). This effect was already present under standard conditions but was much more pronounced under oxidative stress conditions (treatment with 50 *μ*M sodium nitroprusside (SNP)) accompanied by mitochondrial dysfunction [24].

### 3.2. Parallel Effects of Piracetam on Mitochondrial Dynamics

**Figure 4:**
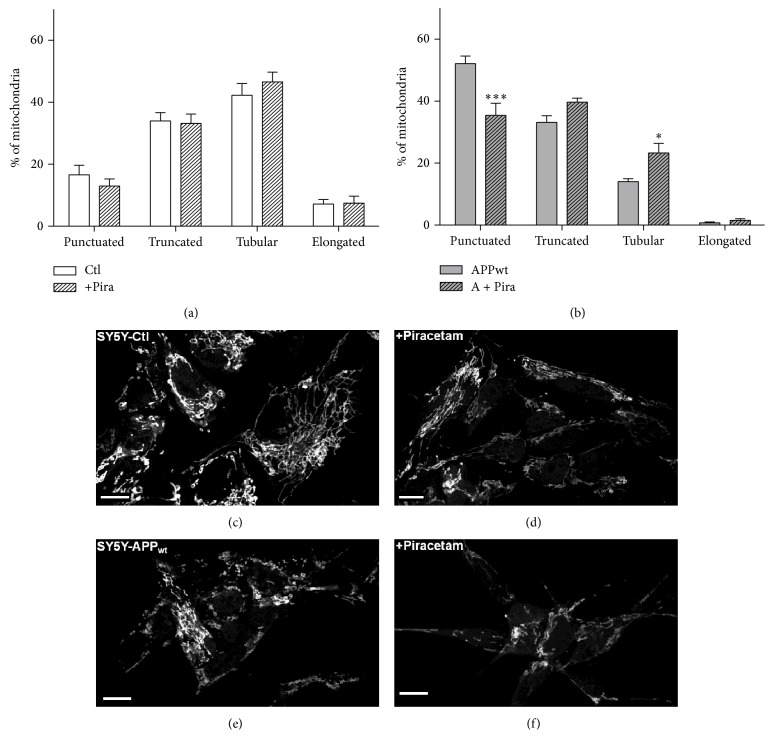
Piracetam improves mitochondrial dynamics in a cell model of early sporadic Alzheimer's disease. (a) Under normal conditions, piracetam (Pira) shows no effect in SH SY5Y Ctl cells but (b) shifts mitochondrial fragmentation induced by an additional APP copy back to baseline conditions. SH-SY5Y cells were seeded and incubated for one day. Afterwards, incubation was started with 1 mM piracetam (Pira). After 72 hours, cells were stained and fixed for analysis of mitochondrial dynamics. ((c), (d)) Representative pictures of SH-SY5Y Ctl and ((e), (f)) SH-SY5Y APPwt cells treated with piracetam; scale bar represents 10 *µ*m. Data are represented as mean ± SEM; Two-Way ANOVA with Bonferroni's posttest (^*∗*^ *p* < 0.05, ^*∗∗∗*^ *p* < 0.001 compared to untreated control cells); *n* = 7.

**Figure 5:**
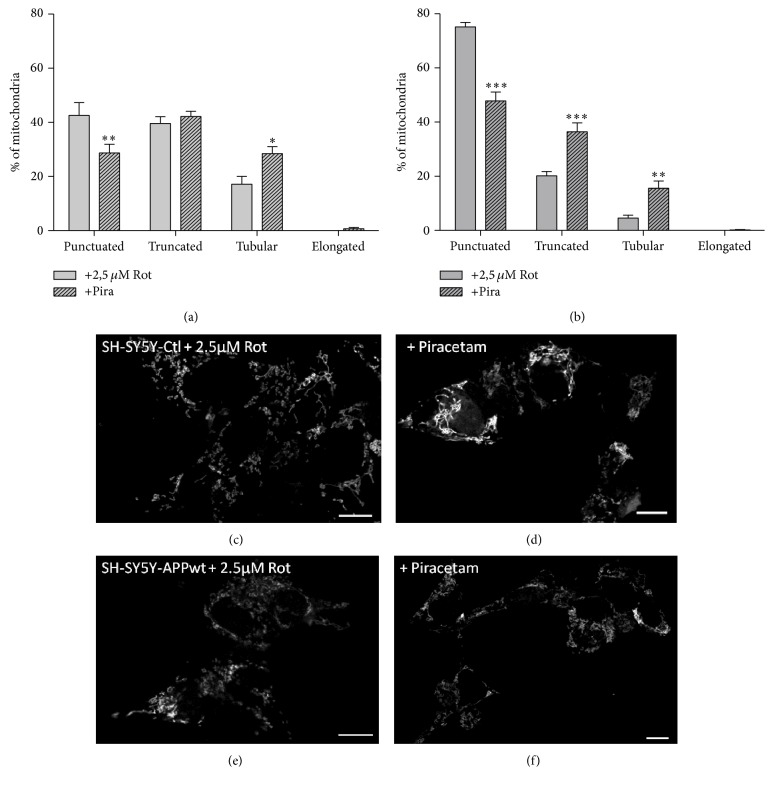
Piracetam ameliorates mitochondrial fission induced by complex I inhibition. Rotenone induces mitochondrial fragmentation in (a) SH-SY5Y Ctl as well as (b) SH-SY5Y APPwt cells. This impairment can be reduced by piracetam (Pira). SH-SY5Y cells were seeded and incubated for one day. Afterwards, incubation was started with 1 mM piracetam (Pira) for 72 hours. During the last 24 hours, 2.5 *µ*M rotenone was added. Afterwards, cells were stained and fixed for analysis of mitochondrial dynamics. ((c), (d)) Representative pictures of SH-SY5Y Ctl and ((e), (f)) SH-SY5Y APPwt cells treated with rotenone and/or piracetam (Pira); scale bar represents 10 *µ*m. Data are represented as mean ± SEM; Two-Way ANOVA with Bonferroni's posttest (^*∗*^ *p* < 0.05, ^*∗∗*^ *p* < 0.01, and ^*∗∗∗*^ *p* < 0.001 compared to rotenone treated cells); *n* = 7.

Mitochondrial dynamics, meaning the ability of mitochondria to undergo changes in size and form, are gaining more and more attention as an important factor regulating mitochondrial function [38] and as mechanism of mitochondrial quality control [34]. Even if reports are sometimes controversial, in most cases, mitochondrial fragmentation is accompanied by reduced mitochondrial function and vice versa [39]. Accordingly, shorter mitochondria are energetically unfavorable. We have previously used confocal microscopy of fixed mitochondria as a very reliable method to analyze mitochondrial dynamics and therefore used this approach to further characterize piracetam's effects on mitochondrial function [29, 30]. Under control conditions, when the equilibrium of mitochondrial dynamics is mainly shifted to the longer shape, piracetam had no significant effect on fission and fusion balance of SH-SY5Y Ctl cells (Figures 4(a), 4(c), and 4(d)). Impairing mitochondrial function either by complex I inhibition, elevated A*β* levels, or the combination of both had a pronounced effect on mitochondrial dynamics with a large shift to the fission site with small mitochondria (Figures 4(b), 4(e), 4(f), and 5). Incubating SH-SY5Y cells for all three conditions with piracetam shifts mitochondrial shape towards elongated forms (>10 *μ*m) and reduces the number of punctuated (<2 *μ*m) mitochondria, which goes in parallel with the positive effect of piracetam on neuritogenesis (Figures 2 and 3). Even when dynamics were substantially impaired as in the cells mirroring the advanced state of LOAD (elevated A*β* and complex I inhibition), piracetam significantly shifted back dynamics to the fusion state with larger mitochondria.

### 3.3. The Possible Role of the Mitochondrial Transition Permeability Pore (mPTP)

**Figure 6:**
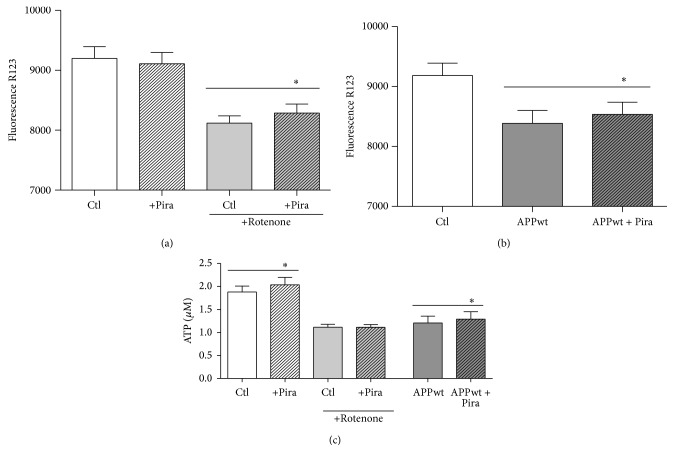
Mitochondrial membrane potential and ATP level are slightly elevated in piracetam treated cells. Mitochondrial membrane potential was analyzed in (a) SH-SY5Y Ctl and (b) SH-SY5Y APPwt cells using the fluorescent dye Rhodamine 123 (R123). In SH-SY5Y Ctl cells, mitochondrial membrane potential is unaltered under normal conditions but decreased after complex I inhibition induced by rotenone. In SH-SY5Y APPwt cells, the mitochondrial membrane potential is decreased and is slightly compensated by piracetam (Pira). SH-SY5Y cells were seeded and incubated for one day. Afterwards, incubation was started with 1 mM piracetam (Pira) for 24 hours. After 30 minutes, 2.5 *µ*M rotenone was added. Finally, mitochondrial membrane potential was determined. (c) Piracetam (Pira) slightly elevates ATP levels under normal conditions in SH-SY5Y Ctl and SH-SY5Y APPwt cells but has no effect in rotenone treated cells. SH-SY5Y cells were seeded and incubated with 1 mM piracetam (Pira) for 72 hours. During the last 24 hours, 2.5 *µ*M rotenone was added. Finally, ATP levels were determined. Data are represented as mean ± SEM; Student's paired *t*-test (^*∗*^ *p* < 0.05 compared to untreated control cells or rotenone treated cells, resp.); *n* = 4–6.

**Figure 7:**
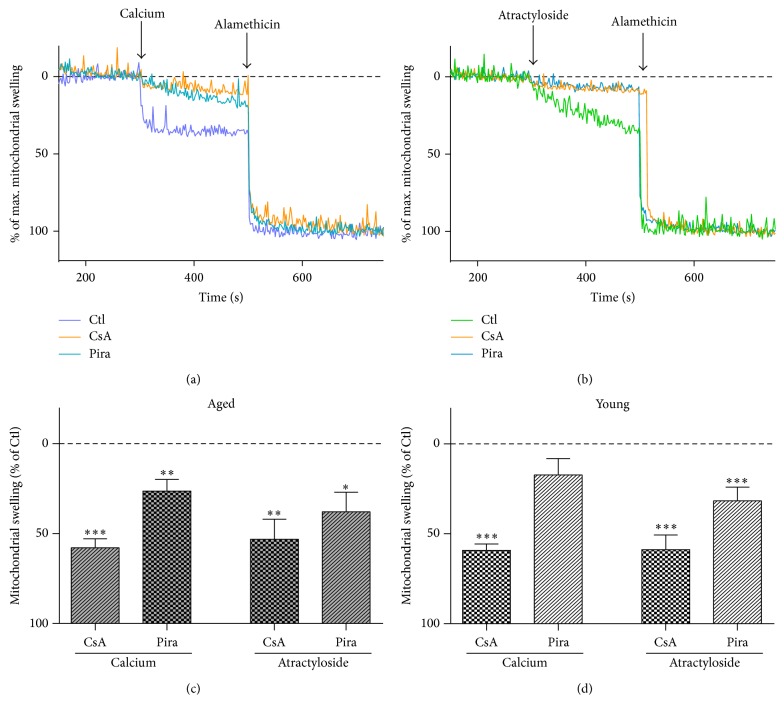
The metabolic enhancer piracetam reduces mPTP opening in aged mice. Mitochondrial permeability transition pore (mPTP) opening was induced by ((a), (c), and (d)) 1 *μ*Mol/mg protein calcium or ((b), (c), and (d)) 400 *μ*M atractyloside, maximal swelling was induced by alamethicin, and changes in absorbance by 540 nm were examined. Mitochondrial swelling after 5 min preincubation with piracetam (Pira, 1 mM) was analyzed using isolated mitochondria from (c) aged (18 months) and (d) young (12 weeks) NMRI mice. Cyclosporine A (CsA, 1 *μ*M) was used as a positive control for the inhibition of mPTP opening. Data are expressed as mean ± SEM; Student's paired *t*-test (^*∗*^ *p* < 0.05, ^*∗∗*^ *p* < 0.01, and ^*∗∗∗*^ *p* < 0.001 compared to mitochondria without CsA or Pira pretreatment); *n* = 4–8.

**Figure 8:**
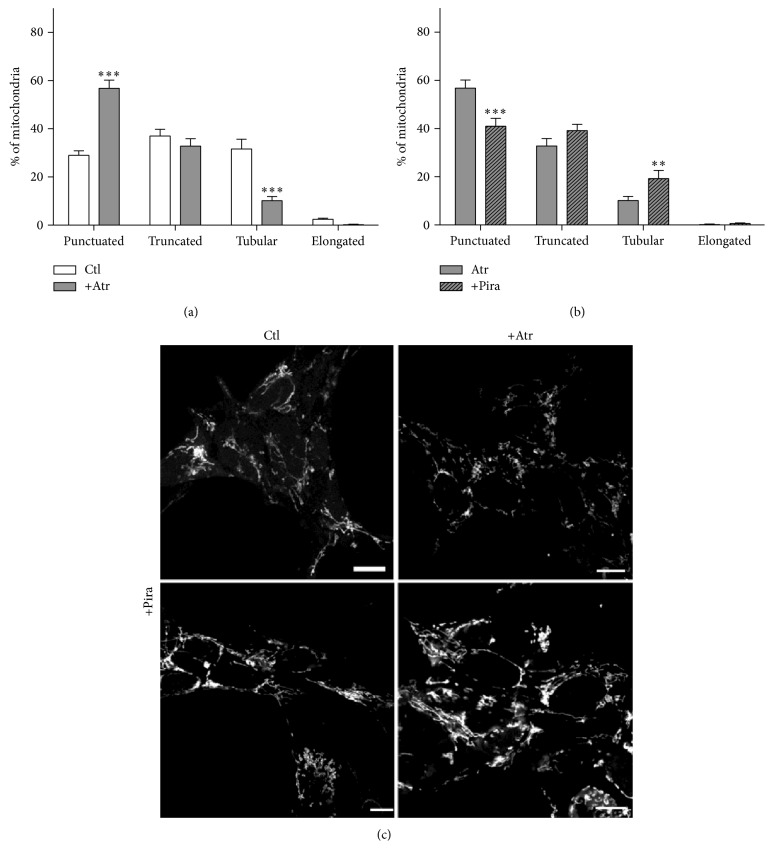
The mPTP inductor atractyloside (Atr) induces mitochondrial fragmentation. (a) Atr induces mitochondrial fragmentation. Cells treated with Atr exhibit more punctuated and less tubular mitochondria. (b) Piracetam (Pira) is able to ameliorate these changes in mitochondrial dynamics. SH-SY5Y Ctl cells were incubated with piracetam (1 mM) for 3 hours, Atr was added for the last 2.5 hours, and cells were stained and fixed for analysis of mitochondrial dynamics. (c) Representative pictures of SH-SY5Y cells treated with media as control, atractyloside (Atr, 100 *μ*M), and/or piracetam (Pira, 1 mM). Scale bar represents 10 *μ*m. Data are represented as mean ± SEM; Two-Way ANOVA with Bonferroni's posttest (^*∗∗*^ *p* < 0.01, ^*∗∗∗*^ *p* < 0.001 compared to untreated control cells or Atr treated cells, resp.); *n* = 5.

We originally assumed that the beneficial effect of piracetam on mitochondria might mainly be associated with elevated ATP levels and increases of the mitochondrial membrane potential [24, 25]; both the effects of piracetam we observed on both parameters for the SH-SY5Y cells (Figure 6) were rather small in comparison with the previous observations using different cell models [24, 25]. However, these small beneficial effects have to been seen in relation to the similar small effects of experimentally induced oxidative stress on both parameters in SH-SY5Y cells [29]. Nevertheless, other mechanisms might be involved. Interestingly, piracetam has been shown to reduce mPTP opening induced by simvastatin [40]. Accordingly, we speculated that inhibition of the mitochondrial permeability transition pore (mPTP) [41–43] might also be involved in the effects of piracetam on mitochondrial dynamics and finally neuritogenesis. In order to examine whether this mechanism of action is also a conceivable explanation for our observations obtained with piracetam, we measured mitochondrial swelling of isolated brain mitochondria by tracking spectrophotometric alterations in light scattering. This method is very useful to analyze mitochondrial permeability transition opening induced by typical inductors like calcium ions or atractyloside. Cyclosporin A (CsA) is a specific inhibitor of mPTP function and serves as a positive control. In these experiments, isolated brain mitochondria of young and old mice were incubated* ex vivo* with 1 mM piracetam. Swelling was induced by calcium chloride (1 *μ*mol/mg protein) or atractyloside (400 *μ*M) and alamethicin was used to detect maximal swelling. In young and aged mitochondria, both conditions induced comparative swelling which could be inhibited by cyclosporine. Similarly, piracetam also reduced swelling substantially but somewhat weaker than cyclosporine (Figure 7). Antagonism of mPTP opening by piracetam was more pronounced in aged than in young mitochondria especially for swelling induced by calcium. In order to possibly link the antagonism of mPTP by piracetam with its effects on mitochondrial dynamics, we assessed the effect of the mPTP opener atractyloside on mitochondrial dynamics. We were able to show a a pronounced shift to smaller mitochondria indicating enhanced fission (Figure 8(a)). Again, piracetam reduced enhanced fission by partially shifting back the equilibrium to the fusion site, probably indicating a direct effect of piracetam on the mPTP (Figure 8(b)).

## 4. Discussion

Impaired cerebral glucose utilization in brain regions like hippocampus and entorhinal cortex represents one of the earliest biomarkers of Alzheimer's disease (AD), detectable long before its clinical manifestation [1, 2]. While other mechanisms also play a role, impaired mitochondrial function seems to be a major cause [1, 2, 11]. Moreover, a large number of findings indicate that nearly all risk factors of AD converge at the level of impaired mitochondrial function [8, 11]. These observations led to the hypothesis that impaired mitochondrial function, associated with reduced energy production and enhanced oxidative stress, emerges by a cascade of specific and nonspecific events finally leading to the severe synaptic and neuronal defects typical for AD [1, 2]. As most important aspect of this mitochondrial cascade hypothesis, the beginning of AD goes back to the very early stages of mitochondrial dysfunction caused by the combined effect of oxidative stress due to aging and slightly elevated *β*-amyloid (A*β*) levels caused by genetic and individual risk factors, long before A*β* deposits begin to form. Since all strategies to remove A*β* plaques have failed so far to lead to symptomatic reductions or even a slowdown of the progression of AD [2, 44, 45], investigating the early phase of mitochondrial dysfunction has become a major strategy to develop new disease-modifying treatments. Based on our and others' previous findings, the metabolic enhancer piracetam could serve as a prototype of compounds able to improve mitochondrial dysfunction over the whole spectrum of the mitochondrial cascade hypothesis of AD [1] from brain aging to the advanced stages of LOAD [18, 24–26].

There are only few cellular or animal disease models reflecting the early phase, especially with respect to the only slight elevation of intracellular A*β* levels, combined with enhanced oxidative stress due to mitochondrial complex I dysfunction in aging. To overcome this deficit, we have previously shown that SH-SY5Y cells can be used to investigate synaptic dysfunction associated with impaired neuritogenesis over a broad range of the aging spectrum, from the normal situation with very low amyloid-*β* levels, to cells with impaired complex I function as a model for the aging process. Furthermore, SH-SY5Y cells bearing an additional copy of the human amyloid-*β* precursor protein (APPwt) gene and therefore producing slightly more A*β* relative to Mock cells [31] can be used as a model for people at risk for late onset AD (LOAD). Finally, initiating complex I dysfunction (as an artificial model of aging) in the APPwt transgenic cells allows us to mirror the very early phase of the initiation of AD* in vitro* [29]. In line with data from PC12 cells where piracetam improved synaptic plasticity following oxidative stress [25], piracetam had no effect on neuritogenesis in SH-SY5Y cells under baseline conditions but substantially enhanced neuritogenesis following complex I inhibition as well as in SH-SY5Ywt cells as a model for early LOAD. The small increase of neurite length in SH-SY5Y APPwt relative to control cells has been explained by elevated levels of the neurotrophic sAPPalpha, although mitochondrial function and dynamics are already impaired in these cells [29]. The concentration of piracetam used in this study (1 mmol/L) has been demonstrated in previous studies to give maximum mitochondrial protection in several different experiments [24, 25, 28] and is quite well within the plasma or tissue concentration range seen with patients or healthy volunteers as well as animals treated with effective doses of piracetam [46–49].

We and others have previously reported that mitochondrial dysfunction and/or impaired synaptic plasticity and neuritogenesis was paralleled by profound changes of mitochondrial dynamics, shifting the equilibrium between mitochondrial fission and fusion to the fission site [29, 50, 51]. This was confirmed for the SH-SY5Y cells also used in this study for all different conditions outlined above by exhibiting a larger percentage, relative to control cells, of small punctuated mitochondria after complex I inhibition or APP expression compared to control cells [29].

Accordingly, we investigated possible effects of piracetam on synaptic deficits and plasticity associated with mitochondrial deficits typical for aging and AD. In agreement with the former assumptions, our data indicate that piracetam improves neuroplasticity over the whole aging-LOAD spectrum as indicated by substantial effects on neuritogenesis. Quite interestingly, piracetam had no effect on neuritogenesis under control conditions without impairment of mitochondrial function. This parallels many other findings regarding piracetam showing beneficial effects mainly when brain function was impaired at the biochemical, physiological, and functional (cognitive) level [18, 52]. In parallel with its effects on improving synaptic plasticity, piracetam also improved mitochondrial dynamics by shifting the fission and fusion balance back to the fusion site with normalized numbers of long (fused) mitochondria. Again, piracetam beneficial effects were mainly seen for the cell models of impaired neuronal function (aging, very early, and advanced LOAD).

After demonstrating pronounced effects of piracetam on mitochondrial dynamics by shifting back the perturbed equilibrium from fission to the energetically more favorable fusion site, being in line with positive effects on mitochondrial membrane potential and substantially improved neuritogenesis, we were interested in the possible target of piracetam's effects at the mitochondrial level. One major player in regulating mitochondrial dynamics is the permeability transition pore (mPTP), a multiprotein-complex of the mitochondrial inner and outer membranes of still unknown defined structure [53, 54]. While the role of mPTP in the mitochondrial fission and fusion balance is still seen controversially, most data suggest that mPTP inhibition is associated with elevated fusion [55–57]. Moreover, some preliminary previous observations suggested that piracetam can inhibit calcium [58] and simvastatin [40] induced mPTP opening where the latter effect seems to be independent of simvastatin's effects on cholesterol metabolism. In line with these findings are our observations that piracetam inhibits mPTP opening induced by calcium as well as atractyloside in mouse brain mitochondria in a rather similar fashion as cyclosporine, despite being to a somewhat lesser extent. Thus, piracetam's effects seem also to be directly associated with the mPTP function. Fitting in our finding with piracetam effect on neuritogenesis as well as mitochondrial dynamics, its inhibitory efficacy was somewhat better in aged mitochondria.

To prove that fission and fusion are regulated by mPTP function in the SH-SY5Y cells used in this study, we investigated the effect of atractyloside on mitochondrial dynamics. Activation of mPTP opening by atractyloside was paralleled by enhanced fission which again could be reduced by piracetam, indicating again that the effects of piracetam on fission and fusion balance seem to be mediated by inhibition of mPTP function. Considering the important role of mitochondrial dynamics for many mechanisms dependent on mitochondrial function like neuroplasticity including neuritogenesis, our data suggest that interference with mPTP function might be an important aspect of piracetam beneficial effects on impaired brain function.

The mechanisms of this effect are not yet finally understood. One possibility could be enhanced fluidity of brain membranes in the presence of piracetam [52, 59, 60], an effect which again is mainly seen when mitochondrial membrane fluidity is reduced by aging or other pathomechanisms [42, 61, 62]. Fluidity of the mitochondrial membrane represents one of the many factors regulating mPTP function. However, findings regarding how membrane fluidity modulates mPTP function are quite complex, but it seems that there is a normal range associated with optimum mPTP closing [63]. Changing fluidity above or below this range leads to mPTP opening events as in case of the aging-LOAD continuum (see above). These changes will be reversed by piracetam's effects on mitochondrial membrane fluidity [42, 61]. Another possible mechanism of piracetam is related to our recent findings with the structurally related antiepileptic levetiracetam, which quite similar to piracetam improves neuritogenesis and mitochondrial dynamics probably by inhibition of mPTP opening [30].

Levetiracetam binds with high affinity to SV2a, a synaptic protein, which however is also located in rather high levels at brain mitochondrial membranes [30]. The affinity of piracetam to this site is rather weak, with an pKi of about 300 *µ*mol/L [64], but considering the therapeutical plasma levels of piracetam which are in this range [46, 47], we cannot rule out that interaction with mitochondrial located SV2a protein may also play a role for the beneficial effects of piracetam on mitochondrial functions reported in the present study.

In summary, our findings reported in the present communication show that inhibition of mPTP function seems to be associated with piracetam's beneficial effects on mitochondrial dynamics and impaired neuritogenesis.
